# Use of BRFSS Data and GIS Technology for Rapid Public Health Response During Natural Disasters

**Published:** 2008-06-15

**Authors:** James B Holt, Ali H Mokdad, Earl S Ford, Eduardo J Simoes, George A Mensah, William P Bartoli

**Affiliations:** Centers for Disease Control and Prevention; Centers for Disease Control and Prevention, Atlanta, Georgia; Centers for Disease Control and Prevention, Atlanta, Georgia; Centers for Disease Control and Prevention, Atlanta, Georgia; Centers for Disease Control and Prevention, Atlanta, Georgia; Northrop Grumman Information Technology, Atlanta, Georgia

## Abstract

Having information about preexisting chronic diseases and available public health assets is critical to ensuring an adequate public health response to natural disasters and acts of terrorism. We describe a method to derive this information using a combination of data from the Behavioral Risk Factor Surveillance System and geographic information systems (GIS) technology. Our demonstration focuses on counties in states that are within 100 miles of the Gulf of Mexico and the Atlantic Ocean coastlines. To illustrate the flexible nature of planning made possible through the interactive use of a GIS, we use a hypothetical scenario of a hurricane making landfall in Myrtle Beach, South Carolina.

## Introduction

The aftermaths of recent natural disasters have highlighted the catastrophic social, economic, and public health impact that these events can have. In December 2004, the Indian Ocean tsunami killed 226,408 people, rendered 1,033,464 homeless, adversely affected an additional 1,356,339, and cost an estimated $7,710,800,000 in damage (1). Between July and October 2005, hurricanes Dennis, Katrina, Rita, and Wilma resulted in the deaths of 1852 people and affected 830,000 more, many of whom became homeless (2).

Although much attention rightly has been given to the immediate safety and acute health needs of these people (3-6), less emphasis has been devoted to the needs, both immediate and long-term, of people with preexisting health conditions. Often, the magnitude of the public health impact is determined by the underlying vulnerabilities of the affected population, including people with chronic diseases, pregnant women, and children, and by the extent of damage to the local public health infrastructure. The public health assets of surrounding communities, which could be used to mitigate damage and provide service to evacuees, also play important roles. Lessons learned from recent disasters suggest that prospective assessment of existing health problems and available resources is essential for effective preparedness and response. Unfortunately, these data are not readily available for most communities at risk.

Hurricane Katrina, which devastated the third most populated metropolitan area on the U.S. Gulf Coast, taught us that this prospective assessment is essential (7). Interruptions in treatment brought on by a disaster increase the risk of death or serious complications for people who require insulin to control their diabetes, for heart attack survivors who take daily clot-preventing medications, for people with severe chronic lung disease who require home oxygen therapy, and for people with kidney failure who are treated with outpatient hemodialysis. Natural disasters often interfere with or totally disrupt the availability of supplemental oxygen supplies. Power outages prevent the use of dialysis and other medical equipment and can exacerbate existing health conditions by preventing the cooling or heating that patients require. Conditions of extreme heat and cold are particularly dangerous for elderly people, pregnant women and their fetuses, neonates, and young children. Lastly, chronic diseases are often aggravated by the lack of food and clean water and the increased levels of physical and mental stress that accompany a disaster (7).

To effectively plan a response to natural disasters, such as hurricanes, floods, and earthquakes, and man-made disasters, such as acts of terrorism, public health officials and first responders need analytic methods to quickly estimate the number of people who will be affected and the subpopulations that are at particular risk. Equally as important is the ability to locate and quantify facilities such as hospitals and schools that are needed during a response. Given the complexity and the sometimes lengthy lead times required for state and local health officials to prepare personnel, facilities, and medical supplies for a public health response, establishing a baseline dataset in advance of a disaster is vital. Preferably, this dataset would be updated frequently and would have the analytic tools needed to model contingencies and develop effective responses, including estimates of the required quantities of essential maintenance medication and treatment for patients with chronic diseases (7).

In the wake of the 2005 hurricanes, Mokdad et al (7) addressed the need for a surveillance tool to support disaster response planning that gives appropriate consideration to people with chronic diseases and other vulnerable populations. Recommendations were that the surveillance tool should have three components: 1) a means of determining the baseline magnitude of the disaster and needs of these vulnerable people, 2) a means of assessing needs and levels of response in an affected area during a disaster, and 3) a means of monitoring the long-term effects of a disaster.

In response to these recommendations, we demonstrate how the Behavioral Risk Factor Surveillance System (BRFSS) and geographic information system (GIS) technology available from Centers for Disease Control and Prevention's (CDC's) National Center for Chronic Disease Prevention and Health Promotion can be combined to meet the need for rapid assessment of subpopulations at risk and to identify available resources in advance of a disaster. We also note the value of the BRFSS in addressing the second and third components of the recommended surveillance tool.

## Data and Technology

We used data from the BRFSS (8-11) to estimate the prevalence of health risk factors and chronic diseases, the 2000 U.S. census (Summary Tape File 3 [SF-3] Long Form) (12) to obtain a sociodemographic baseline, and the American Hospital Association Annual Survey Database to quantify hospital resources (13). Environmental Systems Research Institute, Inc (ESRI) provided data on school locations and attributes by collating data from the U.S. Geographic Names Information System and the U.S. Board of Geographical Names, both of which collect and archive data on civic institutions as part of the U.S. Geological Survey's National Map program (14).

The BRFSS, operated by state health departments with assistance from CDC, collects data on many of the behaviors and conditions associated with the leading causes of morbidity and mortality in the United States. Each month, trained interviewers use an independent probability sample of households with telephones to collect data from the noninstitutionalized population aged 18 years or older. A detailed description of the survey methods is available elsewhere (15). All questionnaires are available online (www.cdc.gov/brfss/questionnaires). We used data from the District of Columbia and the 21 states whose land area partially or completely extends to within 100 miles of the Gulf of Mexico and the Atlantic Ocean coastlines. To ensure that each county-level prevalence estimate was based on a combined sample of at least 50 responses, we combined data from survey years 2001, 2003, 2004, and 2005 (N = 904,531).

BRFSS respondents for the years that we used answered questions pertaining to high blood pressure, use of blood pressure medication, high blood cholesterol, heart attack, heart disease, stroke, diabetes, asthma, and pregnancy. From the answers, we estimated the prevalence of these medical conditions for the general population. We used SAS 9.1.3 (SAS Institute Inc, Cary, North Carolina) and the proc surveymeans design statement to account for the complex sampling design of the BRFSS.

GIS technology has been defined in various ways (16,17), but for succinctness we prefer the definition of Lo and Yeung: "a set of computer-based systems for managing geographic data and using these data to solve spatial problems" (18). For our demonstration, we used ArcGIS 9.2 (Environmental Systems Research Institute, Inc, Redlands, California), which enabled us to merge, analyze, and display data and results in one software application. We obtained GIS shapefiles (i.e., geographic boundary files) of U.S. states and counties (hereafter, *counties* refers to counties and county-equivalents: parishes in Louisiana and independent cities in Virginia) from ESRI, and extracted the coastlines of the Atlantic Ocean and the Gulf of Mexico through GIS-assisted manual editing. The resulting coastline shapefile became the baseline from which we constructed 50- and 100-mile buffers. We chose these radii arbitrarily, as reasonably good markers for the differences in area damage that result from hurricanes of various magnitudes.

## Assessment Techniques

**Figure 1:**
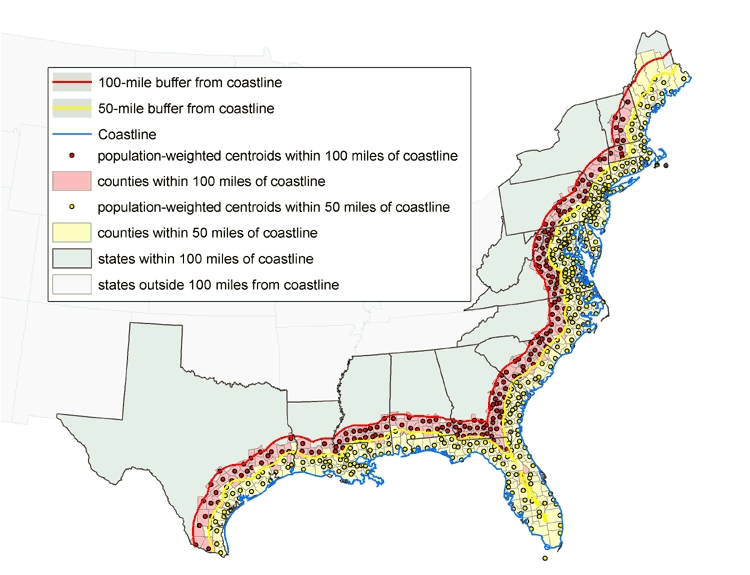
Counties with population-weighted centroids within 50- and 100-mile radius of Gulf of Mexico and Atlantic Ocean coastlines, 2000. Data from U.S. Census Bureau (12).

To estimate the underlying populations at risk within the two buffer zones, we determined which counties the zones comprised. We mapped the population-weighted centroid (center of mass) of the District of Columbia and each county and conducted two spatial joins (a GIS overlay function) between population-weighted centroids and county shapefiles to extract those counties with centroids in both buffer zones (≤50 miles and >50–100 miles from the coastline) (Figure 1). We used population-weighted centroids, which are analogous to centers of gravity, rather than geometric centroids because population-weighted centroids more accurately reflect the spatial distribution and density of county populations.

We imported county sociodemographic data from the 2000 U.S. census (19) into ArcGIS in database format and joined the database to the county shapefile, using county FIPS (Federal Information Processing Standards) codes as the primary join key. The National Institute of Standards and Technology issues a standardized set of numeric codes to ensure uniform identification of geographic entities by all federal government agencies (19,20). These data include variables on total population, age distribution, racial/ethnic distribution, housing units and occupancy status, median housing values, school enrollment by type of school, prevalence of disability by age group, median family income, and prevalence of poverty by age group. We also imported county public health data from the BRFSS into the GIS database. Once the data were joined to the county shapefiles, summary statistics and ratios of the individual variables were computed by area.

**Figure 2:**
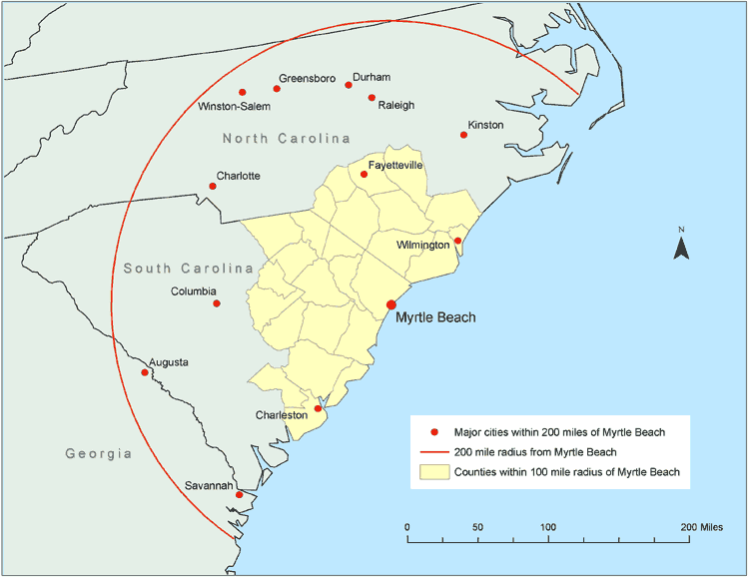
Counties with population-weighted centroids within a 100-mile radius and major cities within a 200-mile radius of Myrtle Beach, South Carolina, 2000. Data from U.S. Census Bureau (12).

To demonstrate the usefulness of a GIS in a real-time emergency, we applied the technology to a hypothetical scenario in which a hurricane makes landfall in the vicinity of Myrtle Beach, South Carolina. We created a 100-mile buffer around the point location for the city of Myrtle Beach and used the GIS to extract those counties with population-weighted centroids within this buffer zone (Figure 2). All values for population demographics, people with chronic diseases, and resources for emergency response were contained within the extracted county-level geographic records in the GIS.

## Sample Assessment

According to the 2000 U.S. census, 139,441,051 people, or approximately 50% of the U.S. population at that time, lived in the total area included in our demonstration (i.e., 21 states and the District of Columbia) (12). Of these people, 66% lived in counties with population-weighted centroids within 100 miles of the Gulf of Mexico and Atlantic Ocean coastlines (57% within ≤50 miles, 9% from >50–100 miles). Note that in our assessment, data for the two coastal buffer zones overlap, so that data for the area in the 100-mile zone include data for the area in the 50-mile zone.

Our assessment shows that approximately 18.2 million people within 100 miles of the coastline were likely to be at particular risk in a disaster because of their age (either <5 years or ≥65 years); approximately 13.8 million, because of being school-aged (i.e., being enrolled in nursery school, kindergarten, or elementary school); and approximately 208,246, because of being inpatients in a hospital (estimated by multiplying the number of hospital beds by a 70% occupancy rate) (Table 1).

**Figure 3:**
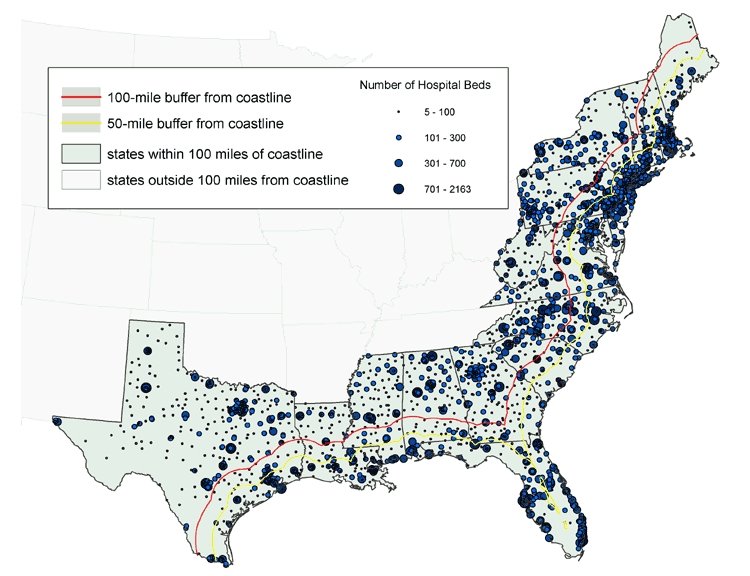
Locations of hospitals, with number of beds per hospital, in states with land area within 100 miles of the coastline. Data from the American Hospital Association (13).

Data joined with the GIS provide the number of hospitals, hospital beds, and hospital workers in total and by state for each zone (Table 2) and the estimated number of people with selected medical conditions in total and by state for each zone (Table 3). By combining the information in Tables 2 and 3, health officials can compare the extent of chronic diseases and the availability of response resources in any coastal area. The number of hospitals in a local area varies greatly throughout each coastal zone, as does the number of beds in a single hospital (Figure 3). As would be expected, areas with large populations tend to have access to greater numbers of hospitals and hospital beds, but the ratio of people to hospitals and of people to hospital beds may actually be lower in highly populated urban areas. This reality underscores the importance of establishing baseline data on the at-risk population and the resources available to respond to surges in demand.

For the Myrtle Beach scenario, an estimated 412,364 people would be at particular risk because of their age; 344,105, because of being in nursery, kindergarten, and elementary schools; and 4661, because of being inpatients in a hospital (Table 4). Given that 16% of people in the area live in poverty, many of these vulnerable people would have to rely on the government for evacuation.

## Flexibility of the BRFSS and GIS

The BRFSS can and has been used to assess needs and levels of response during a disaster and to monitor the long-term effects of a disaster. In response to the unexpected shortfall in the 2004–2005 supply of influenza vaccine, CDC and the Advisory Committee on Immunization Practices (ACIP) recommended prioritizing vaccination for people aged 65 years and older and for others at high risk (21,22). To monitor coverage, the BRFSS added several questions about influenza vaccination, including new questions on priority status and the month and year of vaccination among children and adults (23). Because of the rapid turnaround of BRFSS data, public health officials were able to obtain near–real-time estimates of influenza coverage (24), including county-level estimates based on small-area estimation procedures (25). One study, using data for the New Orleans–Metairie–Kenner, Louisiana, Metropolitan Statistical Area, demonstrated the feasibility of using the BRFSS to estimate baseline information on the number of older adults who may have a disability and thus need assistance in evacuating to shelters or who may need special equipment in the event of a natural disaster (26).

Flexibility is one of the most useful features of a GIS. By altering the planning assumptions that are entered into the GIS, public health officials can conduct analyses quickly and efficiently on any issue for which data are available. Sources could include the National Hospital Ambulatory Medical Care Survey, which has asked questions in the past that may yield data on hospital preparedness for natural disasters and acts of terrorism (27); state-based trauma system registries, which contain data on mass casualties and trauma (28); and CDC's National Center for Health Statistics, which maintains data on the number of live birth deliveries by county, from which estimates can be derived of the number of pregnant women and neonates at a given time. The salient questions for health officials are: What sources of primary data are readily available? To what extent can the surge capacity of identified assets be ascertained reliably? How generalizable are the outputs, and how sensitive are they to the particular type of disaster?

## Figures and Tables

**Table 1:** Selected At-Risk Populations in Gulf of Mexico and Atlantic Ocean coastal zones, by Distance From the Coastline, United States, 2000^a^

**At-Risk Populations**	**Distance from Coastline** b		
	**≤50 miles, No. of People**	**≤100 miles, No. of People**	**>100 miles, No. of People**
**Old and young**	15,807,599	18,204,359	9,049,178
<5 y of age	5,269,967	6,069,337	3,206,434
≥65 y of age	10,537,632	12,135,022	5,842,744
**Below poverty level (%)**	9,585,589 (12.0)	11,409,425 (12.4)	6,402,990 (13.5)
**School-aged population (total)**	21,356,614	24,563,563	12,659,167
Nursery school	1,494,064	1,696,568	829,584
Kindergarten	1,149,218	1,328,574	698,459
Elementary school	9,303,221	10,755,108	5,619,833
High school	4,519,507	5,231,149	2,691,489
College	4,890,604	5,552,164	2,819,802
**Hospital inpatients^c^**	177,787	208,246	117,036

a Data are from the U.S. Census Bureau (12) and the American Hospital Association (13).

b Measured by population-weighted centroids.

c Based on 70% bed occupancy.

**Table 2:** Number of Hospitals and Hospital Beds and Workers in 21 States and the District of Columbia, by Distance From the Coast, United States, 2000^a^

**State or District**	**Distance From Coastline^b^**		
	**≤50 Miles, No.**	**≤100 Miles, No.**	**>100 Miles, No.**
**Total**			
Hospitals	1,189	1,521	1,161
Hospital Beds	253,891	297,494	167,081
Workers	1,313,786	1,529,468	816,505
**Alabama**			
Hospitals	15	35	86
Hospital Beds	2,990	4,626	13,328
Workers	11,357	17,640	59,546
**Connecticut**			
Hospitals	46	47	NA
Hospital Beds	8,862	8,940	NA
Workers	51,430	51,714	NA
**Delaware**			
Hospitals	11	11	NA
Hospital Beds	2,237	2,237	NA
Workers	16,332	16,332	NA
**District of Columbia**			
Hospitals	16	16	NA
Hospital Beds	4,670	4,670	NA
Workers	28,623	28,623	NA
**Florida**			
Hospitals	209	219	NA
Hospital Beds	48,453	50,419	NA
Workers	224,536	230,866	NA
**Georgia**			
Hospitals	19	60	116
Hospital Beds	2,597	7,214	18,558
Workers	12,475	35,940	96,033
**Louisiana**			
Hospitals	102	118	59
Hospital Beds	12,699	14,191	6,229
Workers	59,261	64,342	25,945
**Maine**			
Hospitals	35	39	3
Hospital Beds	3,420	3,542	164
Workers	22,492	23,242	1,423
**Maryland**			
Hospitals	67	70	4
Hospital Beds	13,692	14,131	467
Workers	80,081	82,432	2,395
**Massachusetts**			
Hospitals	92	113	NA
Hospital Beds	19,033	21,758	NA
Workers	122,892	137,682	NA
**Mississippi**			
Hospitals	12	27	80
Hospital Beds	1,892	3,622	10,497
Workers	8,598	16,071	38,048
**New Hampshire**			
Hospitals	18	31	1
Hospital Beds	2,212	3,091	16
Workers	13,447	20,537	100
**New Jersey**			
Hospitals	94	94	NA
Hospital Beds	27,453	27,453	NA
Workers	122,382	122,382	NA
**New York**			
Hospitals	130	142	112
Hospital Beds	44,160	46,251	19,863
Workers	239,885	247,274	105,345
**North Carolina**			
Hospitals	32	58	84
Hospital Beds	5,075	10,063	15,946
Workers	25,086	52,630	88,435
**Pennsylvania**			
Hospitals	85	135	118
Hospital Beds	18,942	27,242	17,960
Workers	99,945	144,892	96,533
**Rhode Island**			
Hospitals	16	16	NA
Hospital Beds	3,293	3,293	NA
Workers	17,748	17,748	NA
**South Carolina**			
Hospitals	24	52	30
Hospital Beds	3,124	7,890	4,155
Workers	16,374	40,408	22,246
**Texas**			
Hospitals	104	150	360
Hospital Beds	17,666	21,557	45,585
Workers	87,908	104,928	212,164
**Vermont**			
Hospitals	NA	6	11
Hospital Beds	NA	376	1,214
Workers	NA	1,933	9,572
**Virginia**			
Hospitals	62	77	37
Hospital Beds	11,421	14,142	6,223
Workers	52,934	68,159	24,508
**West Virginia**			
Hospitals	NA	5	60
Hospital Beds	NA	786	6,876
Workers	NA	3,693	34,212

NA indicates not applicable.

a Data are from the American Hospital Association (13).

b Measured by population-weighted centroids.

**Table 3:** Estimated Numbers of People With Selected Medical Conditions in 21 states and the District of Columbia, by Proximity to the Gulf of Mexico and Atlantic Ocean Coastlines^a^

**State, District**	**Distance From Coastline^b^**	

	**≤50 Miles**	**≤100 Miles**
**Total**		
High blood pressure	2,181,000	2,639,000
Taking blood pressure medication	1,271,000	1,532,000
High blood cholesterol	2,120,000	2,740,000
Heart attack	2,328,000	2,787,000
Heart disease	2,577,000	3,067,000
Stroke	1,489,000	1,773,000
Diabetes	662,000	801,000
Asthma	998,000	1,177,000
Pregnancy	113,000	130,000
**Alabama**		
High blood pressure	19,000	32,000
Taking blood pressure medication	13,000	23,000
High blood cholesterol	15,000	28,000
Heart attack	26,000	41,000
Heart disease	15,000	29,000
Stroke	11,000	24,000
Diabetes	5,000	10,000
Asthma	7,000	11,000
Pregnancy	1,000	2,000
**Connecticut**		
High blood pressure	67,000	67,000
Taking blood pressure medication	48,000	48,000
High blood cholesterol	68,000	68,000
Heart attack	87,000	87,000
Heart disease	113,000	113,000
Stroke	44,000	44,000
Diabetes	21,000	21,000
Asthma	40,000	40,000
Pregnancy	4,000	4,000
**Delaware**		
High blood pressure	21,000	21,000
Taking blood pressure medication	14,000	14,000
High blood cholesterol	19,000	19,000
Heart attack	28,000	28,000
Heart disease	31,000	31,000
Stroke	17,000	17,000
Diabetes	5,000	5,000
Asthma	8,000	8,000
Pregnancy	1,000	1,000
**District of Columbia**		
High blood pressure	15,000	15,000
Taking blood pressure medication	11,000	11,000
High blood cholesterol	18,000	18,000
Heart attack	13,000	13,000
Heart disease	13,000	13,000
Stroke	14,000	14,000
Diabetes	6,000	6,000
Asthma	11,000	11,000
Pregnancy	1,000	1,000
**Florida**		
High blood pressure	494,000	505,000
Taking blood pressure medication	289,000	295,000
High blood cholesterol	412,000	431,000
Heart attack	653,000	676,000
Heart disease	718,000	744,000
Stroke	393,000	403,000
Diabetes	172,000	178,000
Asthma	229,000	238,000
Pregnancy	29,000	29,000
**Georgia**		
High blood pressure	28,000	59,000
Taking blood pressure medication	13,000	32,000
High blood cholesterol	17,000	48,000
Heart attack	21,000	56,000
Heart disease	22,000	46,000
Stroke	18,000	47,000
Diabetes	7,000	16,000
Asthma	9,000	20,000
Pregnancy	1,000	2,000
**Louisiana**		
High blood pressure	67,000	75,000
Taking blood pressure medication	47,000	54,000
High blood cholesterol	52,000	57,000
Heart attack	80,000	85,000
Heart disease	91,000	101,000
Stroke	55,000	60,000
Diabetes	29,000	32,000
Asthma	35,000	38,000
Pregnancy	3,000	3,000
**Maine**		
High blood pressure	39,000	39,000
Taking blood pressure medication	19,000	19,000
High blood cholesterol	36,000	36,000
Heart attack	42,000	42,000
Heart disease	39,000	39,000
Stroke	22,000	22,000
Diabetes	12,000	12,000
Asthma	22,000	22,000
Pregnancy	2,000	2,000
**Maryland**		
High blood pressure	153,000	163,000
Taking blood pressure medication	98,000	103,000
High blood cholesterol	188,000	192,000
Heart attack	169,000	174,000
Heart disease	168,000	174,000
Stroke	98,000	101,000
Diabetes	54,000	55,000
Asthma	93,000	95,000
Pregnancy	10,000	10,000
**Massachusetts**		
High blood pressure	120,000	146,000
Taking blood pressure medication	73,000	91,000
High blood cholesterol	116,000	140,000
Heart attack	155,000	203,000
Heart disease	151,000	193,000
Stroke	83,000	106,000
Diabetes	33,000	41,000
Asthma	73,000	88,000
Pregnancy	6,000	7,000
**Mississippi**		
High blood pressure	9,000	27,000
Taking blood pressure medication	7,000	17,000
High blood cholesterol	12,000	23,000
Heart attack	13,000	36,000
Heart disease	14,000	39,000
Stroke	12,000	24,000
Diabetes	4,000	10,000
Asthma	5,000	10,000
Pregnancy	1,000	2,000
**New Hampshire**		
High blood pressure	18,000	22,000
Taking blood pressure medication	11,000	15,000
High blood cholesterol	27,000	35,000
Heart attack	29,000	36,000
Heart disease	35,000	43,000
Stroke	17,000	23,000
Diabetes	7,000	9,000
Asthma	11,000	15,000
Pregnancy	1,000	1,000
**New Jersey**		
High blood pressure	244,000	244,000
Taking blood pressure medication	148,000	148,000
High blood cholesterol	288,000	288,000
Heart attack	233,000	233,000
Heart disease	282,000	282,000
Stroke	139,000	139,000
Diabetes	64,000	64,000
Asthma	91,000	91,000
Pregnancy	10,000	10,000
**New York**		
High blood pressure	267,000	283,000
Taking blood pressure medication	152,000	165,000
High blood cholesterol	346,000	361,000
Heart attack	254,000	266,000
Heart disease	292,000	314,000
Stroke	201,000	207,000
Diabetes	83,000	87,000
Asthma	132,000	140,000
Pregnancy	19,000	19,000
**North Carolina**		
High blood pressure	81,000	130,000
Taking blood pressure medication	39,000	68,000
High blood cholesterol	58,000	120,000
Heart attack	61,000	110,000
Heart disease	59,000	114,000
Stroke	41,000	79,000
Diabetes	22,000	42,000
Asthma	25,000	52,000
Pregnancy	3,000	7,000
**Pennsylvania**		
High blood pressure	225,000	357,000
Taking blood pressure medication	102,000	166,000
High blood cholesterol	152,000	456,000
Heart attack	119,000	224,000
Heart disease	138,000	247,000
Stroke	82,000	134,000
Diabetes	48,000	84,000
Asthma	82,000	129,000
Pregnancy	7,000	10,000
**Rhode Island**		
High blood pressure	23,000	23,000
Taking blood pressure medication	17,000	17,000
High blood cholesterol	26,000	26,000
Heart attack	27,000	27,000
Heart disease	31,000	31,000
Stroke	15,000	15,000
Diabetes	7,000	7,000
Asthma	13,000	13,000
Pregnancy	1,000	1,000
**South Carolina**		
High blood pressure	61,000	100,000
Taking blood pressure medication	28,000	53,000
High blood cholesterol	42,000	88,000
Heart attack	42,000	86,000
Heart disease	37,000	77,000
Stroke	30,000	62,000
Diabetes	13,000	27,000
Asthma	13,000	28,000
Pregnancy	2,000	4,000
**Texas**		
High blood pressure	99,000	149,000
Taking blood pressure medication	65,000	93,000
High blood cholesterol	93,000	134,000
Heart attack	146,000	201,000
Heart disease	157,000	216,000
Stroke	102,000	135,000
Diabetes	38,000	51,000
Asthma	44,000	59,000
Pregnancy	6,000	7,000
**Vermont**		
High blood pressure	NA	5,000
Taking blood pressure medication	NA	2,000
High blood cholesterol	NA	4,000
Heart attack	NA	4,000
Heart disease	NA	4,000
Stroke	NA	2,000
Diabetes	NA	1,000
Asthma	NA	2,000
Pregnancy	NA	1,000
**Virginia**		
High blood pressure	131,000	172,000
Taking blood pressure medication	77,000	95,000
High blood cholesterol	135,000	163,000
Heart attack	130,000	154,000
Heart disease	171,000	207,000
Stroke	95,000	113,000
Diabetes	32,000	41,000
Asthma	55,000	65,000
Pregnancy	5,000	6,000
**West Virginia**		
High blood pressure	NA	5,000
Taking blood pressure medication	NA	3,000
High blood cholesterol	NA	5,000
Heart attack	NA	5,000
Heart disease	NA	10,000
Stroke	NA	2,000
Diabetes	NA	2,000
Asthma	NA	2,000
Pregnancy	NA	1,000

NA indicates not applicable.

a Data are from the Behavioral Risk Factor Surveillance System (8-11).

b Measured by population-weighted centroids.

**Table 4:** Selected At-Risk Populations and Available Resources Within 100-mile Radius of Myrtle Beach, South Carolina^a^

**Community Characteristics**	**No. ≤100 Miles From Coastline^b^**
**At-Risk Populations**	
**Total population**	2,244,538
<5 y of age	153,529
≥65 y of age	258,835
**Below poverty level (%)**	359,126 (16.0)
**School-aged children (total)**	597,453
Nursery school	39,054
Kindergarten	34,130
Elementary school	270,921
High school	131,082
College	122,266
**High-risk adults**	443,000
High blood pressure	94,000
Taking blood pressure medication	20,000
High blood cholesterol	76,000
Heart attack	73,000
Heart disease	69,000
Stroke	51,000
Diabetes	28,000
Asthma	30,000
Pregnant	2,000
**Available resources**	
**Schools**	1,067
**Hospitals**	43
Hospital beds	6,658
Hospitalizations (70% bed occupancy)	4,661
Hospital workers	38,118

a Data are from the Behavioral Risk Factor Surveillance System (8-11), the U.S. Census Bureau (12), and the American Hospital Association (13).

b Measured by population-weighted centroids.
